# Antioxidant capacity, flavor and physicochemical properties of FH06 functional beverage fermented by lactic acid bacteria: a promising method to improve antioxidant activity and flavor of plant functional beverage

**DOI:** 10.1186/s13765-022-00762-2

**Published:** 2023-01-28

**Authors:** Xian-Tao Yan, Ziqi Zhang, Yubao Wang, Wenmiao Zhang, Longfei Zhang, Yang Liu, Dawei Chen, Wenqiong Wang, Wenlong Ma, Jian-Ya Qian, Ruixia Gu

**Affiliations:** 1 Jiangsu Key Laboratory of Dairy Biotechnology and Safety Control, Yangzhou University, Yangzhou, 225127 People’s Republic of China; 2 Department of Cuisine and Nutrition, Hanshan Normal University, Chaozhou, People’s Republic of China; 3 Tourism College of Zhejiang, Hangzhou, People’s Republic of China

**Keywords:** Antioxidant, Plants, Lactic acid bacteria (LAB), Functional food, Flavor

## Abstract

The ability of natural plants to treat chronic diseases is closely related to their antioxidant function. Lactic acid bacteria (LAB) fermentation is an effective way to improve the nutritional value, biological activity and flavor of food. This study investigated the pH, titratable acidity, total polysaccharide, total flavone, total saponin, total polyphenol, and antioxidant activity of the FH06 beverage before and after probiotic fermentation. Results: After fermentation, FH06 had lower contents of total polysaccharides, total flavonoids, total saponins and total polyphenols but higher titratable acidity. The antioxidant activity was tested by total antioxidant capacity (FRAP method) and DPPH· scavenging ability. The FRAP value significantly increased after fermentation (*P* < 0.05), and the maximum increase was observed for *Lactobacillus fermentum* grx08 at 25.87%. For DPPH· scavenging ability, the value of all fermentations decreased, and *L. fermentum* grx08 had the smallest reduction at 2.21% (*P* < 0.05). The results of GC–MS and sensory analysis showed that fermentation eliminated bad flavors, such as grass, cassia and bitterness, and highlighted the fruit aroma and soft sour taste. Conclusion: The FRAP value and sensory flavor of FH06 fermentation by *L. fermentum* grx08 were significantly improved, indicating its great potential as a functional food with both strong antioxidant activity and good flavor.

**Graphical Abstract:**

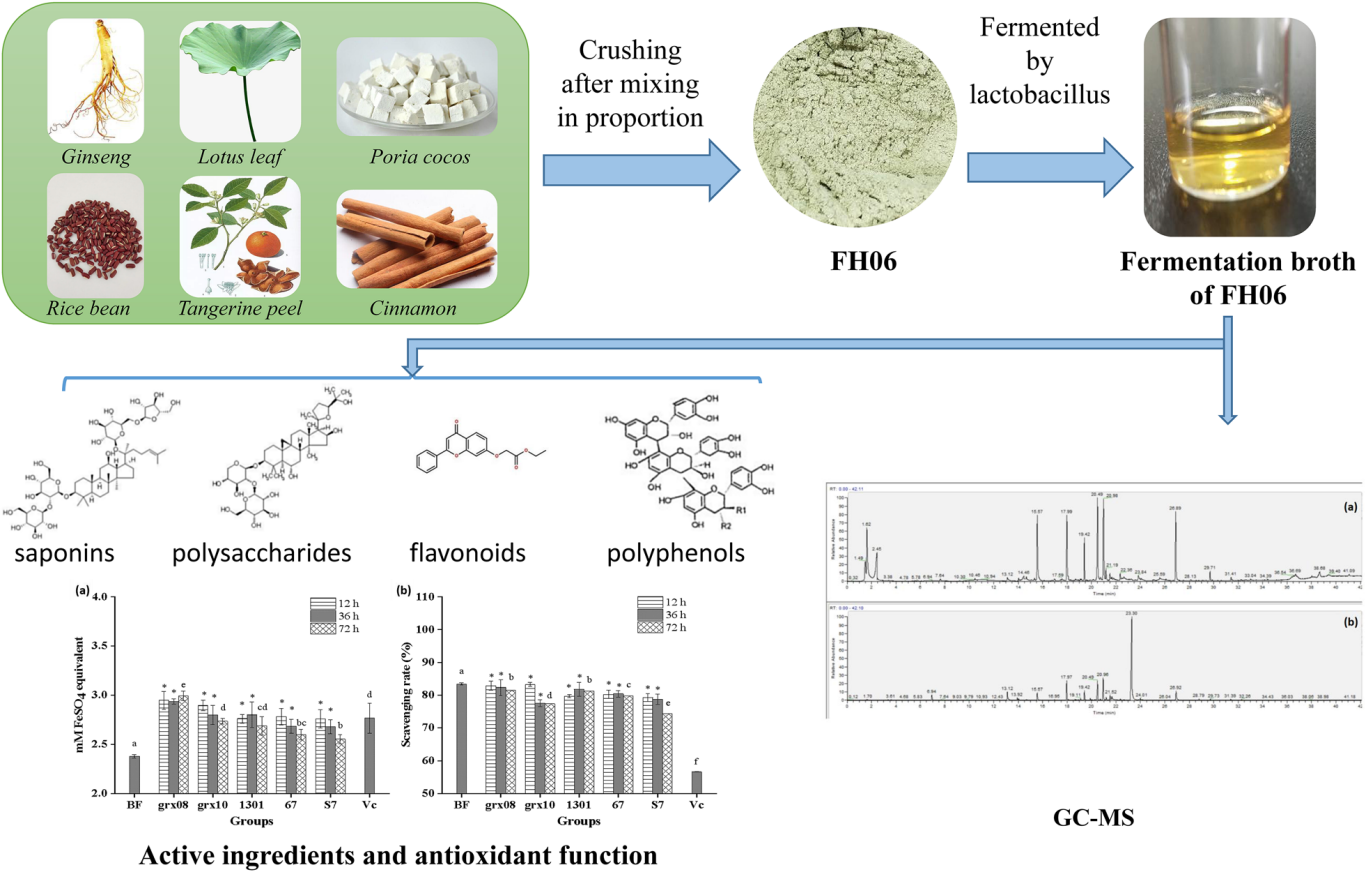

**Supplementary Information:**

The online version contains supplementary material available at 10.1186/s13765-022-00762-2.

## Introduction

Obesity and other chronic diseases are prevalent in the world [1]. Natural plants have important advantages and a long history of application in the prevention and treatment of obesity and other chronic diseases [2]. Oxidative stress and chronic inflammation play a central role in the pathogenesis of obesity, diabetes, chronic nephritis, fatty liver and other chronic diseases [3]. Moreover, oxidative stress can activate inflammatory mediators, leading to the development of metabolic and inflammatory diseases [4]. Studies have shown that the mechanisms of action of many natural plants in the treatment of chronic diseases are closely related to antioxidant function [5]. In China, there is a special class of natural plant raw materials that not only have the nutritional ingredients of ordinary food and can be used as diet but also have the functional ingredients of medicine and can be used as medicine. The National Health Commission of the People’s Republic of China lists it as a raw material for both food and medicine. This kind of special food raw material is traditionally called medicine food homology (MFH) raw material, which has a universal and long history in daily diet and clinical application, so it has a high edible safety [6].

With the development of health concepts, there is an increasing demand for fermentable plant products (FPPs). Chen et al. [7] found that water extract of fermented rice bran (FRB) had strong antioxidant activity and protective effect on liver injury in rats fed with high-fat diet. Hwang et al. [8] found that fermentation by *Leuconostoc mesenteroides* KCCM 12010P improved the antioxidant and anti-inflammatory properties of ginseng. Song et al. [9] found that hydroponic ginseng-fortified Yogurt fermented by *Lactobacillus brevis* B7 can significantly increase the antioxidant activity in RAW 264.7 cells. *Bacillus subtilis-*fermented dried citrus peel extract exerts anti-inflammatory activity in RAW 264.7 macrophages induced by lipopolysaccharide (LPS) [10]. Fermentation of apple juice by *Saccharomyces cerevisiae* and *Lactobacillus plantarum* significantly improved antioxidant activity [11]. At present, there are only studies on the fermentation of individual raw materials, such as ginseng, rice bean and tangerine peel, but there are few reports on the use of LAB to ferment plant-based compound beverages with homologous food and medicine to enhance their biological functions. However, there is a consensus that multiple targeted therapies are needed to treat chronic conditions such as obesity. The compound plant-based beverage FH06 is composed of six MFH materials: ginseng, lotus leaf, poria cocos, rice bean, tangerine peel and cassia. Their combination is based on the traditional Chinese medicine weight loss theory of tonifying qi and invigorating the spleen, dispelling dampness and resolving phlegm [12]. Ginseng [13], lotus leaf [14], poria cocos [15], tangerine peel [16], rice bean [17] and cassia [18] all have antioxidant and anti-inflammatory properties and are commonly used as food or dietary supplements for weight loss in China, Korea and other eastern countries.

In addition, the conditioning of chronic diseases such as obesity is not a short-term process, as is its formation. Therefore, the flavor of products is an important factor for consumers to adhere to long-term use. Some natural plant raw materials generally have bad flavors, such as grass and bitterness [19], so it is difficult for consumers to persist in consumption. Studies have found that microbial fermentation can reduce the odor of plants such as grass [20]. Therefore, this study focuses on screening LAB strains from probiotics of longevity people who are suitable for growing in FH06, improving efficacy and improving flavor to develop functional beverages with both efficacy and good flavor. The growth was characterized by the number of viable bacteria. The physicochemical properties were characterized by pH, titration acidity, total polysaccharides, total flavonoids, total saponins and total polyphenols. The antioxidant activity was evaluated by DPPH· scavenging activity and total antioxidant capacity (FRAP value). Volatile flavor compounds were determined by gas chromatography–mass spectrometry (GC–MS).

## Materials and methods

### Chemicals

1, 1-Diphenyl-2-trinitrophenylhydrazine (DPPH) was purchased from Shanghai Macklin Biochemical Technology Co., Ltd. Gallic acid, rutin, ginsenoside Re and anhydrous glucose were obtained from Shanghai Yuanye Biotechnology Co., Ltd, China. Folin-Ciocalteu phenol reagent was purchased from Sangon Bioengineering (Shanghai) Co., Ltd, China. The total antioxidant (FRAP) kit was purchased from Nanjing Jiancheng Biological Engineering Research Institute Co., Ltd, China. The rest of the chemicals, reagents, consumables and culture media were purchased from National Pharmaceutical Chemical Reagent Co., Ltd, China.

### Preparation of FH06

Ginseng (*Panax ginseng* C. A. Meyer), lotus leaf (*Nelumbo nucifera* Gaertn.), poria cocos (*Poria cocos* (Schw.) Wolf.), rice bean (*Vigna umbellata* (Thunb.) Ohwi et Ohashi), tangerine peel (*Citrus sinensis* (Linn.) Osbeck) and cassia (*Cinnamomum cassia* Presl) were mixed in the proportion of 10 ∶ 6 ∶ 10 ∶ 10 ∶ 3 ∶ 1 by weight, fully crushed, mixed evenly with ultrapure water in the proportion of 1 ∶ 10 (g/ml), soaked at room temperature for 30 min, heated at 100 °C for 30 min, cooled and centrifuged at 4000 × g for 10 min. The supernatant was FH06. The raw materials above were purchased from Beijing Tongrentang Health Pharmaceutical Co., Ltd., Beijing, P. R. China, and identified by the chief pharmacist Ying Yao, Yangzhou University, P. R. China. Voucher specimens YZU20201015-1 ~ 6 were preserved in the specimen room of Yangzhou University, Yangzhou, Jiangsu Province, P. R. China.

### Preparation of fermented FH06

*Lactobacillus fermentum* grx08 (grx08, CGMCC No: 7695), *Lactobacillus rhamnosus* hsryfm1301 (1301, CGMCC No: 8545), *Lactobacillus rhamnosus* grx10 (grx10, CGMCC No: 2526), *Lactobacillus plantarum* 67 (67, CGMCC No: 21268) and *Lactobacillus plantarum* S7 (S7, CGMCC No: 19021) were provided by the Jiangsu Provincial Key Lab of Dairy Biotechnology and Safety Control, Yangzhou University, China. Lactic acid bacteria strains were inoculated into conventional MRS medium and cultured at 37 °C for 18 h. Then, the cells were centrifuged at 5000 × g for 1 min, the supernatant was poured out, sterile normal saline was added to wash the precipitated bacteria, and washing was repeated twice. Finally, the bacterial suspension was obtained by resuspending bacteria in sterile physiological saline and adjusting the OD_600_ to 1.0. The above bacterial suspension was inoculated into FH06 at a ratio of 3% (v/v) and then cultured under anaerobic conditions at 37 °C for 72 h. After inoculation, 40 mL was sampled immediately, of which 1 mL was used to count living bacteria, and the rest was centrifuged at 5000 × g for 5 min. The supernatant was taken as the fermentation broth for 0 h and stored at − 80 °C until use. After that, samples were obtained and treated according to the above method at 12 h, 36 h and 72 h.

### Determination of live bacterial count, pH and titratable acidity

The number of living bacteria in the sample was determined by the plate colony counting method. The pH value was measured by an FE20 pH meter (METTLER TOLEDO Int. Ltd., Zurich, Switzerland). The pH meter was calibrated with pH 4.01 and 7.00 standard buffer solution before use. The titratable acidity was determined by standard sodium hydroxide (0.1 mol/L) titration with a pH value up to 7.0. The determination method of titration acidity is as follows: Weigh 5 g sample and titrate by 0.1 mol/L NaOH solution until a pH value up to 7.0. The number of milliliters of NaOH solution used × 20 is the titratable acidity value of the sample (°T).

### Determination of total polysaccharide content (TPSC)

The TPSC in the sample was determined by the phenol–sulfuric acid method and slightly modified according to the method of Nazeam et al. [21]. Anhydrous glucose was used as the standard. A total of 0.5 mL of sample (diluted 50 times with distilled water) was added to 0.5 mL of 6% phenol solution, and then 2.5 mL of concentrated sulfuric acid was added and mixed well. Keep at 25 °C for 30 min. Finally, the absorbance at 490 nm was measured by a microplate reader (Thermo Fisher Co., Ltd., Waltham, USA). The data were expressed as mg of glucose equivalent (GlcE) per milliliter of FH06.

### Determination of total flavonoid content (TFC)

The TFC was determined by the aluminum nitrate colorimetric method with rutin as the standard and slightly modified according to the method of Chen et al. [22]. The sample (0.5 mL) was mixed with 0.05 g/mL NaNO_2_ (1 mL) and allowed to stand for 6 min; then, 0.1 g/mL Al (NO_3_)_3_ (1 mL) was added, mixed evenly and allowed to rest for 6 min. Next, 0.04 g/mL NaOH (3 mL) was added, mixed evenly and allowed to rest for 15 min; finally, the absorbance at 510 nm was measured by a microplate reader (Thermo Fisher Co., Ltd., Waltham, USA), and the data are expressed as milligrams of rutin equivalent (RE) per milliliter of FH06.

### Determination of total polyphenol content (TPC)

TPC was slightly modified according to the literature of Derakhshan et al. [23]. As determined by the Folin-Ciocalteu method, a standard curve was prepared with gallic acid as the standard. A total of 400 μL of Flynn-Chocard reagent was added to 100 μL of standard or sample and mixed well. After 1 min, 300 μL of 10% sodium carbonate was added to the mixture, mixed well, brought to a volume of 5 mL with ultrapure water, and incubated at room temperature for 60 min. The absorbance was measured at 765 nm by a microplate reader (Thermo Fisher Co., Ltd., Waltham, USA). Data are expressed as mg of gallic acid equivalent (GAE) per milliliter of FH06.

### Determination of the total saponin content (TSC)

TSC was determined by the vanillin-sulfuric acid colorimetric method with ginsenoside Re as the standard and slightly modified according to the method of [24]. Ten microliters of the sample was placed in a 1.5 mL centrifuge tube and dried at 42 °C for 1 h. Then, 100 μL of vanillin (10% w/v) dissolved in ethanol was added and mixed well. Add 750 μL of 75% concentrated sulfuric acid in an ice bath and mix well. Then, the samples were incubated in a water bath at 60 °C for 20 min. To stop the reaction, the samples were cooled on ice for 10 min. Finally, the absorbance was measured with a 96-well microplate reader (Thermo Fisher Co., Ltd., Waltham, USA) at 544 nm. The data were expressed as mg of ginsenoside Re equivalent (GRE) per milliliter of FH06.

### Determination of total antioxidant capacity (FRAP value)

The determination of the total antioxidant capacity of plasma by the iron reduction (FRAP) method was carried out according to the kit instructions. Then, 180 μL of FRAP working liquid was added to each well of the 96-well plate, and 5 μL of ultrapure water was added to the blank control well. Five microliters of sample were added to the test well and mixed gently. A_593 nm_ was measured after incubation at 37 °C for 3 min. The standard curve was determined with FeSO_4_ as the standard product. The FRAP value of the sample is expressed in mM FeSO_4_ equivalents.

### Determination of scavenging ability of DPPH

The method mentioned in the literature by Kwon et al. was slightly modified [25]. Samples diluted 10 times with 45 µL and 100 μL of DPPH solution at 0.2 mM (soluble in ethanol) were added to a 96-well plate, mixed well, and placed in the dark for 30 min at room temperature. The absorbance A_1_ at 516 nm was measured by a microplate reader (Thermo Fisher Co., Ltd., Waltham, USA), and 45 µL of anhydrous ethanol was used to replace the sample as blank absorbance A_0_. Ascorbic acid was selected as the positive control. The unit of the DPPH· scavenging rate is recorded as ‘%’.

1 $${\text{DPPH}}\cdot{\text{ scavenging rate }}\left( \% \right) \, = \, \left( {{\text{A}}_{0} - {\text{A}}_{{1}} } \right)/{\text{A}}_{0} *{1}00$$

### Determination of volatile flavor compounds

The determination of volatile flavor compounds before and after fermentation was slightly modified according to the method of Dan et al. [26].

Aging of the extraction head: aging occurred at the inlet at 250 °C for 30–60 min. Solid phase microextraction conditions: adsorption on a magnetic stirrer at 50 °C for 45 min. Desorption conditions: desorption for 3 min at 250 °C. The carrier gas was He, with a flow rate of 1.0 mL/min; splitless injection, inlet temperature of 250 °C. Temperature program mode: the starting temperature was 35 °C, and after holding for 5 min, it increased to 140 °C at a rate of 5 °C/min, held for 2 min, increased to 250 °C at a rate of 10 °C/min, and held for 3 min. Full scan mode, EI ion source, electron energy 70 eV, ion source temperature 230 °C, mass scan range m/z: 35–500 AMU, no solvent delay.

The NIST2.2 standard library of the Masshunter workstation that comes with the machine was used to automatically retrieve the mass spectrum data of each component and calculate the relative content of each component with o-chlorodiphenyl as the internal standard.

### Sensory evaluation

According to the sensory scoring criteria (Additional file 2: Table S1), 10 trained graduate students in food science and engineering conducted sensory evaluations on various flavor and mouthfeel indicators of SHLE before and after fermentation, giving 0 to 10 points as quantitative indicators. The taster only knows the number of samples. Normal temperature boiled water was used at the beginning of the evaluation and between the evaluations of the different samples to clean the palate and remove residual taste.

### Statistical analysis

All samples were repeated three times. Statistical analysis was performed using SPSS 22 (Statistical Package for the Social Science, SPSS Ins., Chicago, USA). The results are presented as the means ± SE, and the differences among the different samples were analyzed using one-way analysis of variance (ANOVA, Tukey's Method). Correlation and partial correlation were used to analyze the relationship among different factors. Values of *P* < 0.05 or *P* < 0.01 were considered statistically significant.

## Results and discussion

### Growth of different lactic acid bacteria in FH06

**Fig. 1:**
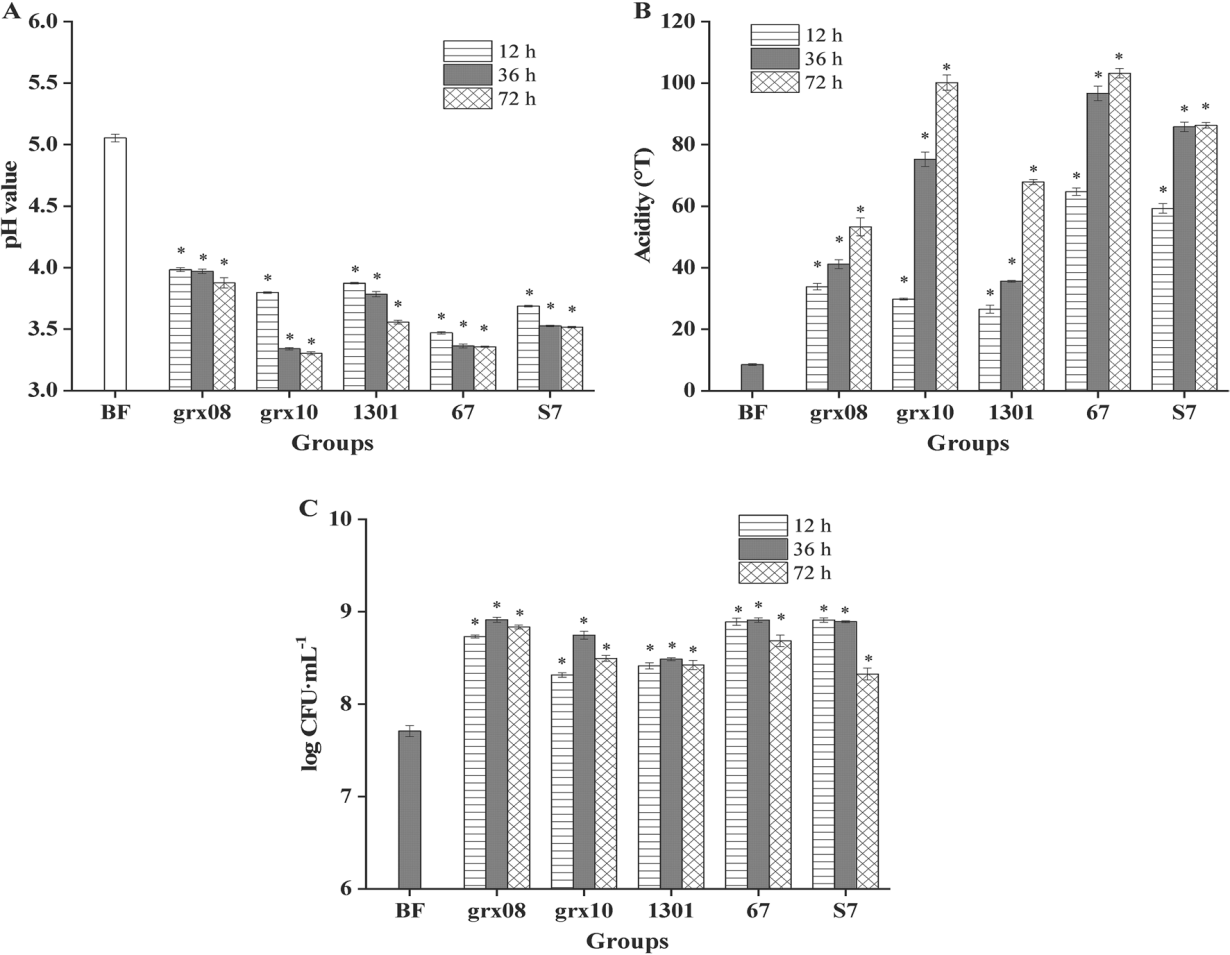
Changes in pH (**A**), acidity (**B**) and viable bacteria count (**C**) of FH06 fermented by different LAB. * represents a significant difference compared with before fermentation (BF) (*P* < 0.05)

The pH, titratable acidity and number of viable bacteria of FH06 fermented at 37 °C for 12 h, 36 h and 72 h are shown in Fig. 1. It can be seen from the graph that six lactic acid bacteria, including grx08, 1301, grx10, 67 and S7, had good growth characteristics in FH06. After fermentation, the pH value of FH06 (Fig. 1A) was significantly lower than that of unfermented FH06 (*P* < 0.01), while the titratable acidity (Fig. 1B) was significantly higher than that of unfermented FH06 (*P* < 0.01). In short, the pH value of FH06 before fermentation was 5.05, and the pH values of different LAB after fermentation were 3.30 (grx10), 3.35(67), 3.51(S7), 3.56(1301) and 3.88(grx08) from low to high. This is similar to the results reported in the literature that the pH value of papaya juice fermented by *L. plantarum* decreased from 5.34 to 3.55 [27]. The pH value of fermentation FH06 decreased significantly, which was attributed to the organic acids produced by fermentation. The titratable acidity of FH06 before fermentation was 8.50°T, and after 72 h of fermentation, the titratable acidity was 103.22°T (67), 100.15°T (grx10), 86.27°T (S7), 67.85°T (1301) and 53.30°T (grx08). The pH value of 67, grx10 and S7 decreased rapidly, and the titratable acidity increased rapidly, but the number of viable bacteria decreased obviously after 36 h (Fig. 1C). Most likely due to faster fermentation and excessive nutrient consumption, the accumulation of metabolites such as organic acids causes LAB to be under the dual stress of starvation and acid, thus inhibiting their growth and reproduction. After 72 h of fermentation, the highest viable cell count was 8.84 lg (CFU/mL) for grx08, and the lowest was 8.33 lg (CFU/mL) for S7.

### Effects of LAB fermentation on some components of FH06

**Fig. 2:**
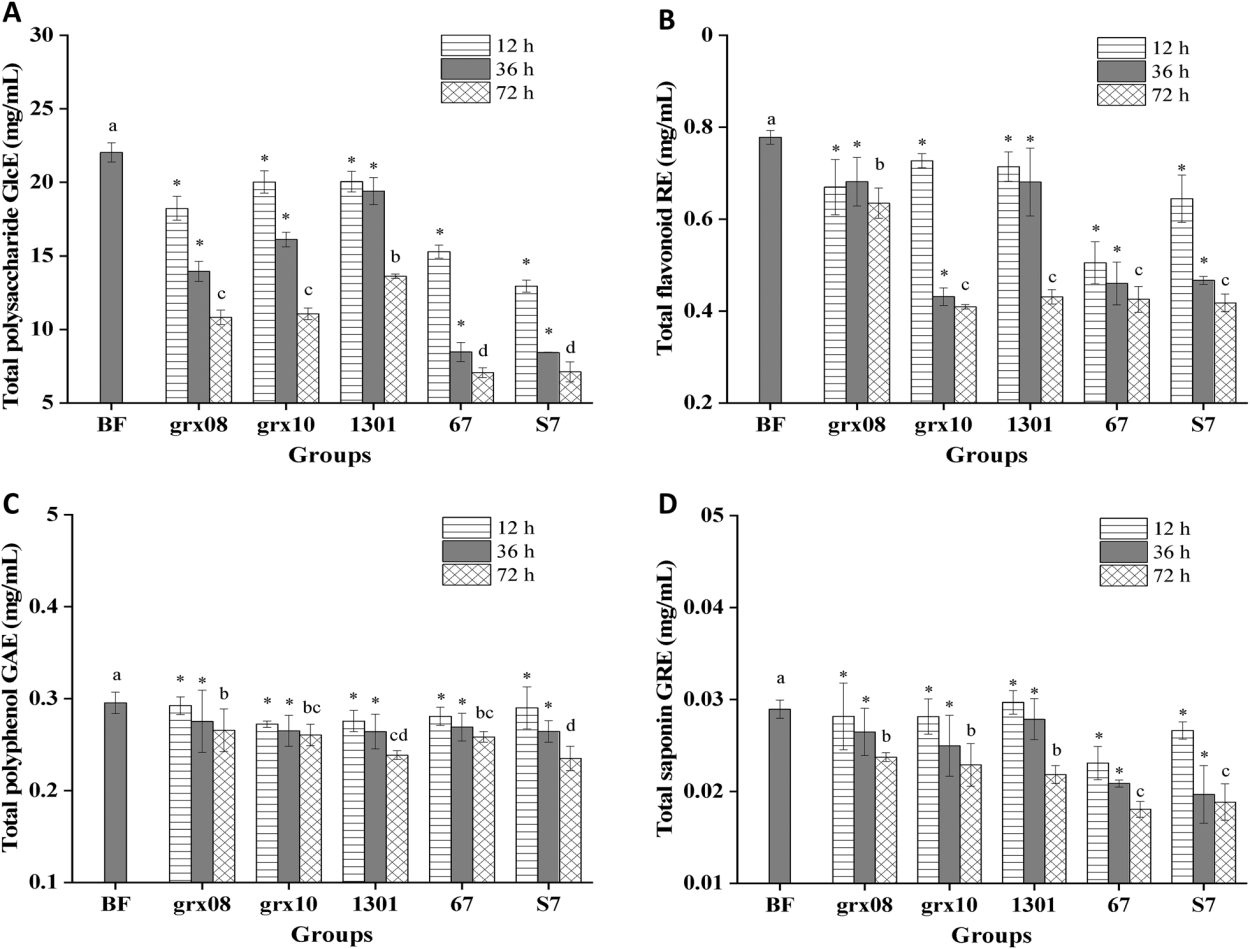
Changes in the contents of functional components in FH06 fermented by LAB. **A** Total polysaccharide; **B** Total flavonoid; **C** Total polyphenol; **D** Total saponin. * Indicates a significant difference from before fermentation (BF) (*P* < 0.05). Different letters indicate significant differences before fermentation (BF) and after fermentation for 72 h *(P* < 0.05). glucose equivalent (GlcE), rutin equivalent (RE), gallic acid equivalent (GAE), ginsenoside Re equivalent (GRE)

The changes in some components of FH06 during 12 h, 36 h and 72 h of fermentation by LAB are shown in Fig. 2. The contents of total polysaccharides, total flavonoids, total polyphenols and total saponins decreased to different degrees (*P* < 0.05).

#### Total polysaccharides

After fermentation by LAB for 72 h, the total polysaccharide content of FH06 decreased the most (Fig. 2A), and the decrease in each LAB group was as follows: 1301 (− 38.21%) < grx10 (− 49.81%) < grx08 (− 50.91%) < S7 (− 67.72%) < 67 (− 67.91%). Of them, *L. plantarum* (67 and S7) consumed much more sugar in speed and quantity than other strains. These results were consistent with the results of previous studies showing that fermentation by *L. plantarum* significantly decreased the reducing sugar content of Bog bilberry juice [28]. The possible reason is that *L. plantarum* releases *β*-glucosidase, which breaks down sugars into their own carbon source for growth [29].

#### Total flavonoids

After 72 h of fermentation, the total flavonoid content of each LAB group (Fig. 2B) decreased from low to high as follows: grx08 (− 18.44%) < 1301 (− 44.63%) < 67 (− 45.32%) < S7 (− 46.34%)) < grx10 (− 47.43%). Tkacz et al. [30] found that a decrease in flavonoids content was observed after fermentation in juices inoculated with *L. plantarum* DSM 100,813 and DSM10492 strains by 8.3 and 10.3%, respectively. In turn, the juice inoculated with the *L. plantarum* DSM 20,174 strain had a final flavonoidconcentration that was 9.5% higher than that of the uninoculated juice. However, *L. plantarum subsp.* had no significant effect on the flavonoids content in sea buckthorn juice. They believed that the type of LAB strain determines the difference in flavonoids concentration. Therefore, studies have also found that LAB fermentation of papaya juice can increase the total flavonoid content [27].

#### Total polyphenols

After 72 h of fermentation, the total polyphenol content decreased the least (Fig. 2C), and the decrease in each LAB group was grx08 (− 10.06%) < grx10 (− 11.8%) < 67 (− 12.52%) < 1301 (− 19.21%) < S7 (− 20.44%). This is consistent with the reported result that the phenolic acid content of seabuckthorn apple juice after 72 h of fermentation by LAB decreased by 17.7% [30]. It was also reported that *Solanum lycopersicum L.* decreased its polyphenol content after in vitro gastrointestinal digestion and colon fermentation [31]. Phenolic acids exist mainly in the form of covalent bonds in plants. The decrease in phenolic acids in the process of FH06 fermentation may be due to the microbial decomposition of the covalently bonded macromolecular phenolic acids into small molecules.

#### Total saponins

After 72 h of fermentation, the total saponin content of each LAB group (Fig. 2D) decreased in the order of grx08 (− 18.04%) < grx10 (− 20.94%) < 1301 (− 24.57%) < S7 (− 34.90%) < 67 (− 37.66%). Park et al. [32] used *Phellinus linteus* for solid fermentation of ginseng. Fermentation increased the total saponin content of the sample. The contents of Rg2, Rc, Rh1(S), Rh1(R) and Rd increased, but the contents of Re and Rf decreased. Lessa O. A [33]. used *Penicillium roqueforti* to produce saponins by solid-state fermentation of cocoa shells. The reason for the increase in the total amount may be that the cellulase produced by fungal solid fermentation destroys the plant cell wall and increases the dissolution of saponins and other substances.

In general, *L. plantarum* (67 and S7) made more use of the above four functional components in different species of LAB. This may be related to the strong ability of *L. plantarum* to ferment carbohydrates [34]. Except for the total polysaccharide, the decrease in the content of the other three functional components was the smallest when grx08 was fermented, which was consistent with the result of the lowest titratable acidity. The utilization of different components by different LAB is different.

### *Effect of LAB fermentation on the antioxidation function of FH06 *in vitro

**Fig. 3:**
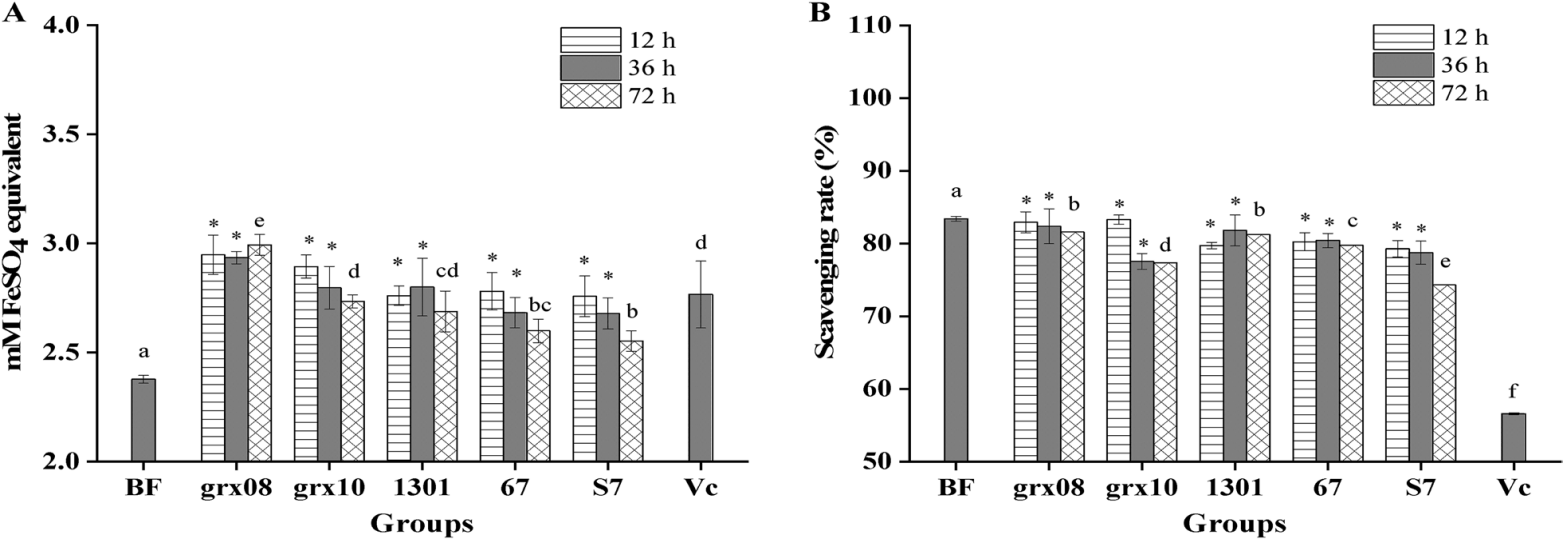
Antioxidant function of different LAB before and after fermentation of FH06. **A** Total antioxidant capacity (FRAP value). **B** DPPH·scavenging ability * indicates a significant difference compared with before fermentation (BF) (*P* < 0.05). Different letters indicate a significant difference compared with BF and VC (0.2 mg/mL) after fermentation for 72 h (*P* < 0.05)

The antioxidant properties of FH06 fermented by LAB for 12 h, 36 h and 72 h are shown in Fig. 3. The total antioxidant capacity (FRAP method) and DPPH· scavenging capacity were tested before fermentation (BF) and 0.2 mg/mL vitamin C (Vc).

**Fig. 4:**
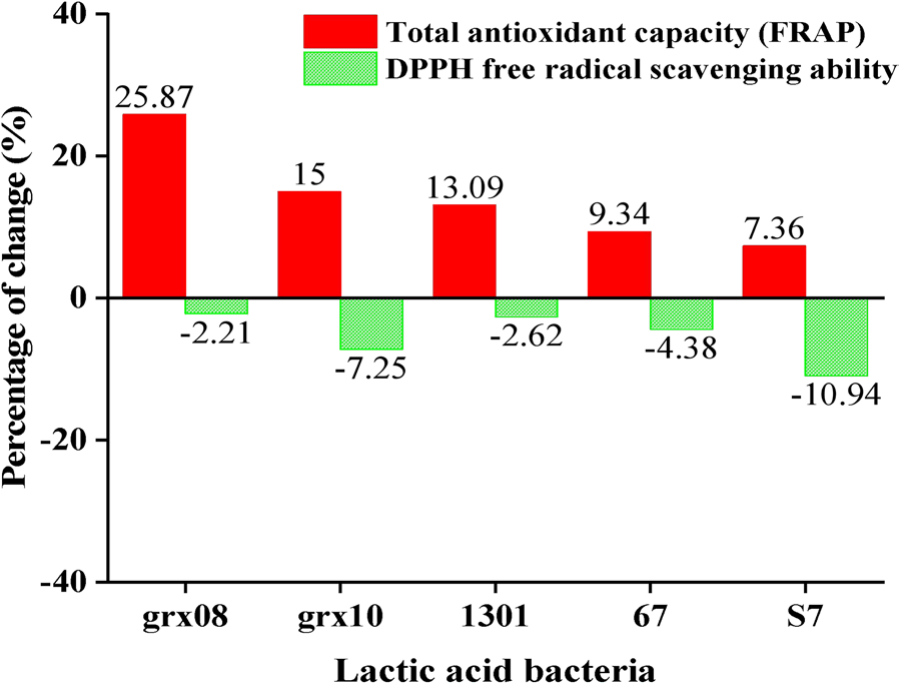
Percentage change of antioxidant properties of FH06 after 72 h fermentation compared with before fermentation

Figure 3A shows that the FRAP value gradually increased after the fermentation of grx08, and the other four LABs showed a trend of first rising and then falling after fermentation. This is consistent with the trend that the FRAP value of *L. acidophilus* fermentation significantly decreased after the 48 h fermentation period [27]. In general, FH06 significantly increased the FRAP value after fermentation by LAB (*P* < 0.05). This is consistent with the report that fermenting quinoa with LAB can improve in vitro antioxidant capacity (FRAP) [35]. Figure 4 shows that the increase in the FRAP value of FH06 by different LAB fermentations was as follows: grx08 (25.87%) > grx10 (15.00%) > 1301 (13.09%) > 67 (9.34%) > S7 (7.36%). After grx08 fermentation, the FRAP value (Fig. 3A) was significantly higher than that of the other fermentation groups and the positive control group (*P* < 0.05).

Oxidative stress and inflammation are closely related pathophysiological processes, one of which is easily induced by the other. Thus, both processes are found in many pathological conditions at the same time [3]. The fermentation process has been shown to enhance the anti-inflammatory activity of herbs through a variety of mechanisms. In addition, there is increasing evidence that enhancing the anti-inflammatory activity of herbal medicines through fermentation is mediated by regulating the gut microbiome [36], so the antioxidant and anti-inflammatory effects in vivo need to be further validated in animal studies.

### *Effects of LAB fermentation on the DPPH*· *scavenging activity of FH06 *in vitro

Figure 3B shows that the scavenging rate of BF was 83.42% before fermentation, and the DPPH· scavenging ability was decreased by fermentation (*P* < 0.05), but the reduction was smaller (Fig. 4) and still higher than 56.61% of the positive control (*P* < 0.05). The results showed that grx08 (− 2.21%) < 1301 (− 2.62%) < 67 (− 4.38%) < grx10 (− 7.25%) < S7 (− 10.94%) decreased the scavenging rate of DPPH·. In general, FH06 before and after fermentation has a strong DPPH· scavenging capacity. In this study, the sample was diluted 10 times and reacted with 0.2 mM DPPH solution at a ratio of 45:100, and the clearance rate was still above 74%. Among them, the DPPH· clearance rate of FH06 remained at 81.57% after grx08 fermentation for 72 h, which is equivalent to 0.32 mg/mL VC. Zhou et al. [37] used LAB to ferment kiwifruit to improve the scavenging ability of DPPH·, which is related to the increased total phenol content. In this paper, the total phenol content and DPPH· scavenging ability after fermentation are slightly reduced. Correlation analysis (Table 1) also showed that DPPH· scavenging ability was positively correlated with total polyphenol content (*P* < 0.05). Similarly, studies such as Chen R. [27]showed that *L. acidophilus-*fermented papaya juice can scavenge DPPH·. The activity was significantly reduced after the fermentation process, the inhibition rate dropped from 81.90% to 55.60%, and its polyphenol content was also reduced by 14.83%.

**Table 1:** Correlation coefficients between antioxidant capacity and some compounds in FH06

r	TPSC	TFC	TPC	TSC	Acidity
TAC	0.139	0.246	0.161	0.318	− 0.191
DPPH	0.608*	0.722**	0.569*	0.591*	− 0.696**
Acidity	− 0.886**	− 0.937**	− 0.587*	− 0.897**	1

r: Pearson’s correlation coefficient

^*^P < 0.05

**P < 0.01

Some studies have shown that spathulenol has antioxidant and anti-inflammatory activities and shows high antioxidant activity in the DPPH system, with an IC_50_ value of 85.6 μg/mL [38]. It is speculated that spathulenol (Additional file 1: Fig. S1A, RT: 31.42 min, Additional file 2: Table S3 No. 88) may be a reason for its strong ability to remove DPPH·.

### *Effect of the pH of the fermentation broth of FH06 on its antioxidant function *in vitro

FH06 is acidic after being fermented by LAB, and its pH value drops below 4.0. To remove the influence of pH value, the supernatant group before (0 h, Con) and after fermentation (72 h) of each group was uniformly adjusted to a pH value of 7.0 with saturated NaOH solution, and then the antioxidant function characteristics of each treatment group (pH treatment) were measured.

#### The effect of pH on the FRAP value

**Fig. 5:**
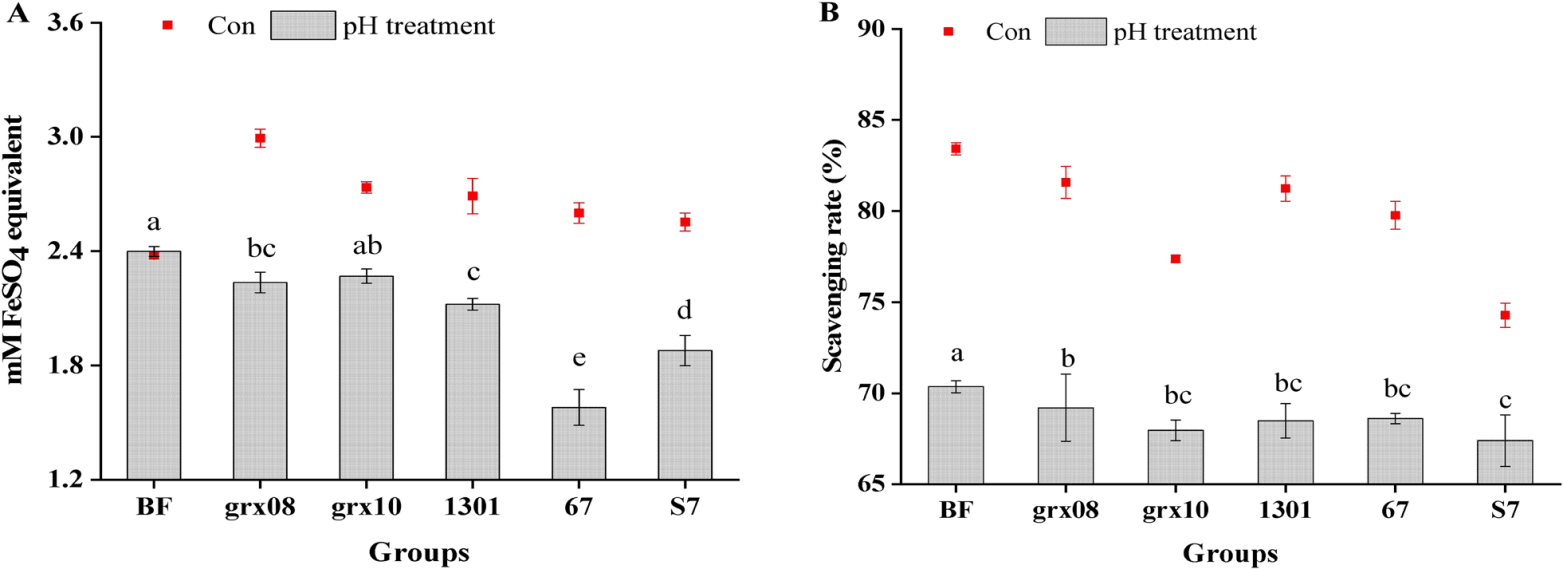
Comparison of the antioxidant properties of the FH06 extract before and after adjusting the pH to 7.0. **A** Total antioxidant capacity (FRAP value); **B** DPPH·scavenging rate. Data are expressed as the mean ± standard deviation (SEM). "Con" means the supernatant stock before and after fermentation; "pH treatment" refers to adjusting the supernatant stock before and after fermentation with a saturated NaOH solution to a pH of 7.0. Note: different letters indicate significant differences among groups after pH adjustment (*P* < 0.05)

Figure 5A shows that after adjusting the pH, the FRAP value of each fermentation group decreased significantly. It may be that fermentation consumes part of the antioxidant active substances as nutrients and converts them into antioxidant active acids. These acidic substances (H^+^) are neutralized by OH^−^ in NaOH, thus reducing the antioxidant capacity. Studies have shown that the organic acid content of fruit vinegar is significantly positively correlated with antioxidant activity and may be an active substance that exerts antioxidant activity. This shows that the improvement in antioxidant capacity (FRAP) is closely related to the production of organic acids.

#### The effect of pH on the scavenging ability of DPPH

Figure 5B shows that after adjusting the pH value to 7.0, the DPPH· scavenging capacity of each group (pH treatment) decreased significantly before and after fermentation. It may be that some active substances were destroyed during the addition of NaOH, thus reducing the DPPH· scavenging ability.

### Principal component analysis

**Fig. 6:**
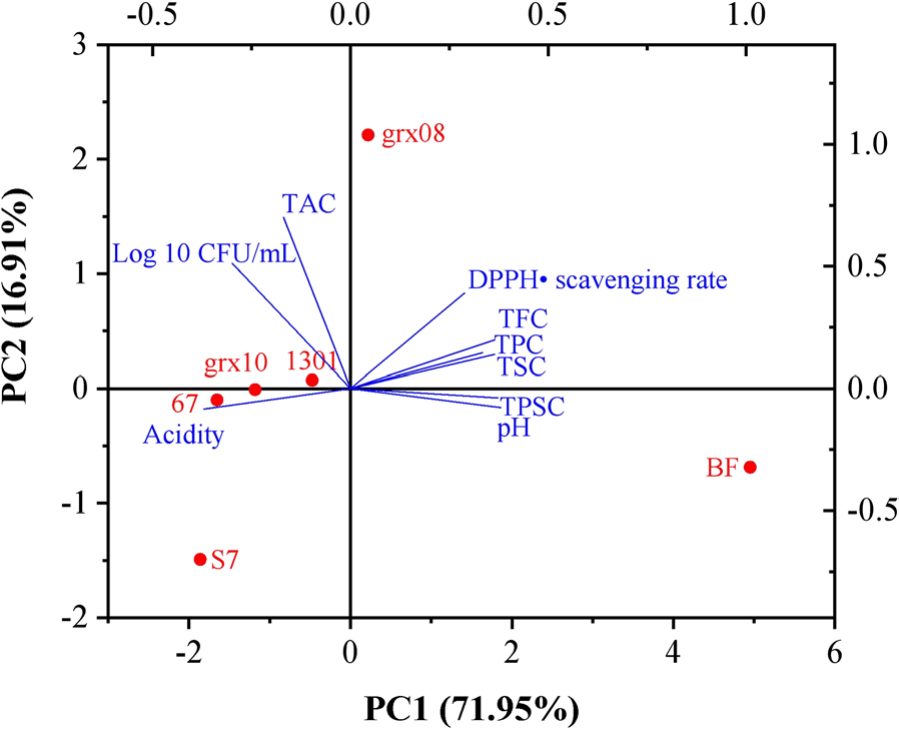
Principal component analysis of fermentation growth, fermentation characteristics and antioxidant properties of different LAB

Principal component analysis (Fig. 6) can clearly distinguish BF and post fermentation samples. FH06 changed significantly after LAB fermentation, and the total polysaccharide, total flavonoids, total polyphenols, total saponins, DPPH· clearance rate and pH decreased after fermentation. In contrast, the titratable acidity, viable bacteria and FRAP value showed an upward trend. Among them, *L. fermentum* grx08 had the smallest reduction in overall active substances after fermentation, the largest increase in FRAP value (25.87%), and the smallest reduction in DPPH· scavenging capacity (− 2.21%). It can be seen that *L. fermentum* grx08 is a good candidate strain for fermenting FH06 functional beverages to improve its antioxidant properties.

### Effect of fermentation on the FH06 sensory score

Lactic acid bacteria fermentation significantly changed the sensory score of FH06 (Additional file 2: Table S2). Fermentation significantly reduced the smell of cassia, grass and bitterness of FH06 (*P* < 0.05). In contrast, fermentation increased the sour taste and fruity flavor (*P* < 0.05). There was no significant difference among the different lactic acid bacteria in the changes in green grass, bitterness and cassia flavors (*P* > 0.05). However, there were differences in the changes in sour taste and fruit flavor among the different strains (*P* < 0.05). Grx08 and 1301 had higher scores on palatable sour taste and fruit flavor. The total score of sensory evaluation of FH06 fermented by grx08 was the highest. Therefore, the fermentation broth of grx08 was selected for the determination of volatile substances.

### Effect of L. fermentum grx08 fermentation on volatile flavor compounds of FH06

The GCMS total ion current diagram before and after fermentation of FH06 is shown in Additional file 1: Fig. S1, which shows that fermentation has a greater impact on volatile substances. A total of 91 volatile flavor substances were detected before and after fermentation (Additional file 2: Table S3). The types of volatile substances before and after fermentation were 63 and 55, respectively. The volatile components of each component were searched through the NIST11 standard library, mainly including esters, alkenes, acids, aldehydes, ketones, phenols and alcohols. The odor activity value (OAV) is mainly used to evaluate the contribution of individual volatile compounds in food to the overall aroma. Volatiles with an OAV value higher than 1 are generally considered to have a significant contribution to the overall aroma. Before fermentation, 16 volatile compounds had OAVs greater than 1, including 2 esters, 1 ketone, 8 aldehydes, and 5 alcohols (Table 2). After fermentation, only 2 volatile compounds had OAV values greater than 1, including an ester and an alcohol. The most obvious changes after fermentation are aldehydes. The 8 aldehydes with OVA values > 1 only have one remaining after 72 h of fermentation. Among them, cinnamaldehyde (Additional file 2: Table S3, No. 57) is the main flavor substance in FH06. It has a clear cassia flavor before fermentation, which is not easy to accept as a beverage, but after fermentation, the cassia flavor is not distinguished from the senses. The GCMS results showed that after fermentation, its content decreased from 3402.57 μg/L to 7.55 μg/L, and the OAV value decreased from 4.54 to 0.01 (Table 2, No. 6). Therefore, it is no longer a key substance of flavor after fermentation.

**Table 2:** Key flavor substances and their OAV values in FH06 before and after fermentation

No.	Volatile components	Odor threshold(μg·L^−1^)	OAVs	
			Before fermentation	After fermentation
	Ester compounds			
1	Methyl methylanthranilate	0.25	1295.3553	884.019
2	Methyl anthranilate	7.00	1.0933	0.5556
Subtotal	2		2	1
	Ketone compounds			
3	Carvone	27.00	1.4166	0.7604
Subtotal	1		1	0
	Aldehyde compounds			
4	Butanal, 3-methyl-	0.35	33.8091	–
5	Butanal, 2-methyl-	1.00	7.2550	–
6	Cinnamaldehyde	750.00	4.5368	0.0101
7	Hexanal	73.00	2.3619	–
8	Benzene acetaldehyde	6.30	2.1045	–
9	Heptanal	4.10	1.8485	–
10	Pentanal	12.00	1.4900	–
11	Nonanal	8.00	1.2701	–
Subtotal	8		8	0
	Alcohol compounds			
12	Linalool	0.22	2181.1849	1042.4713
13	4-Heptenal, (Z)-	0.03	155.6933	–
14	1-Octen-3-ol	1.50	43.3660	0.4485
15	1-Butanol, 3-methyl-	4.00	3.3923	–
16	1-Hexanol	5.60	1.9257	–
Subtotal	5		5	1
Total	16		16	2

“-” means not detected

There were 5 kinds of alcohols with OVA values > 1 before fermentation, and only 1 kind was left after fermentation. Sensory, FH06 has a clear grassy and astringent taste before fermentation, and these undesirable flavors disappear after fermentation. Studies have shown that n-hexanol and n-hexanal have astringent taste and grassy smell [39]. The GCMS results showed that n-hexanol (Table 2, No. 16) and n-hexanal (Table 2, No. 7) were not detected after fermentation.

After fermentation, the main key flavor substances are linalool (OAV value = 1042.47) and methyl N-methylanthranilate (OAV value = 884.02). However, compared with BF, the content of the above two is reduced. However, the reduction or disappearance of undesirable flavor substances such as cinnamaldehyde, n-hexanal and n-hexanol can highlight the flavor of linalool and methyl N-methylanthranilate. Linalool is the main component of many essential oils, and its smell is described as floral, citric acid, fresh and sweet [40]. Linalool represents more than 70% of floral terpenoids [41]. It has been reported that linalool is one of the main contributors to aromatic compounds in papaya fruit. Methyl N-methylanthranilate is an important aromatic compound in citrus peel oil [42]. It is an important fruit flavor substance and is widely used in the preparation of orange oil, peach, grape and other flavors. Therefore, fermented FH06 has a pleasant fruity aroma.

In addition, FH06 before fermentation has obvious bitterness in the senses, but the bitterness disappears after fermentation. Studies have found that the bitter taste of Compositae plants can be reduced by adjusting the pH value to acidity [43]. In this study, it may be that fermentation produced organic acids, which lowered the pH of the beverage and masked the bitter taste. It is also possible that fermentation has metabolized bitter substances [44]. Due to the higher threshold of acids, their influence on volatile aromas is not obvious. However, in terms of taste, it adds a soft sour taste to the FH06 beverage.

The COVID-19 pandemic has made consumers generally raise their health awareness, but the requirements for the taste and flavor of the products have also remained high. In general, FH06 fermented by *L. fermentum* grx08 produced beneficial changes in taste and flavor, removing undesirable flavors such as grass, bitter and cassia, and increasing fruity aroma and soft sourness.

## Conclusion

In this study, the plant-based composite beverage FH06 was used as the substrate to select suitable fermentation strains from human-derived probiotics. Probiotic fermentation significantly affects the physicochemical properties and antioxidant capacity (FRAP value) of FH06 by changing the pH, titratable acidity, total polysaccharides, total flavonoids, total saponins and total polyphenol content of FH06. Among them, the FRAP value of FH06 fermented by *the L. fermentum* grx08 strain was significantly higher than that of unfermented FH06 and FH06 fermented by other LAB. GCMS and sensory analysis of FH06 fermented by the grx08 strain showed that fermentation removed the original green grass, cassia, bitter and astringent flavors and highlighted the fruit aroma and soft sourness, making fermented FH06 more acceptable to consumers. It is more conducive for consumers to insist on drinking for a long time. In short, the research results show that the FH06 beverage fermented by probiotics has the potential to become a functional food with both strong antioxidant activity and good flavor. In the future, the identification of its biologically active compounds and their metabolic pathways, shelf-life testing, and extensive evaluation of the health-promoting effects of fermented FH06 beverages will all be valuable supplements to these studies.

## Supplementary Information

**Additional file 1: Fig. S1:** GC–MS total ion chromatogram before and after fermentation (A) before fermentation (B) after fermentation.

**Additional file 2: Table S1.** Sensory Scoring Criteria. **Table S2.**Sensory Score Results.** Table S3.** Changes in the relative mass concentration of volatile flavor compounds before and after fermentation.

## Data Availability

All data generated or analysed during this study are included in this published article.
