# Supplementary material for: Antioxidant capacity, flavor and physicochemical properties of FH06 functional beverage fermented by lactic acid bacteria: a promising method to improve antioxidant activity and flavor of plant functional beverage
Source: Appl Biol Chem. 2023 Jan 28;66(1):7. doi: 10.1186/s13765-022-00762-2 (PMC9883607; doi:10.1186/s13765-022-00762-2)
Supplement: Supplementary file 2 — Additional file 2: Table S1. Sensory Scoring Criteria. Table S2.Sensory Score Results. Table S3. Changes in the relative mass concentration of volatile flavor compounds before and after fermentation. [file 13765_2022_762_MOESM2_ESM.docx]

**Table S1** Sensory evaluation standard

| Evaluation indicators | evaluation standard | Score |
| --- | --- | --- |
| Grassy (10 points) | No grass smell | 7-10 |
|  | Has a faint grassy smell | 4-6 |
|  | Obvious grassy smell | 0-3 |
| Bitterness (10 points) | No bitterness | 7-10 |
|  | Has a slight bitter taste | 4-6 |
|  | Bitterness is obvious | 0-3 |
| Cassia (10 points) | No cassia flavor | 7-10 |
|  | Has a light cassia flavor | 4-6 |
|  | Strong cassia flavor | 0-3 |
| Sour taste (10 points) | Moderately sour | 7-10 |
|  | Excessive or mild acidity | 4-6 |
|  | No sour taste | 0-3 |
| Fruit flavor (10 points) | Fruit fragrance is obvious | 7-10 |
|  | A light fruity aroma | 4-6 |
|  | No fruit scent | 0-3 |
| Cool feeling (10 points) | Cool feeling is obvious | 7-10 |
|  | Has a slight cool feeling | 4-6 |
|  | No cool feeling | 0-3 |

**Table S2** Sensory Score Results

| Evaluation indicators | BF | grx08 | grx10 | 1301 | 67 | S7 |
| --- | --- | --- | --- | --- | --- | --- |
| Grassy | 1.8±0.98 ^a^ | 8.1±0.7 ^b^ | 6.9±0.83 ^b^ | 7±0.77 ^b^ | 7.1±0.83 ^b^ | 7.2±0.98 ^b^ |
| Bitterness | 2.5±1.2 ^a^ | 7.6±0.92 ^b^ | 7.1±0.94 ^b^ | 7.3±0.78 ^b^ | 7.2±0.75 ^b^ | 7.2±0.87 ^b^ |
| cassia | 3.2±1.33 ^a^ | 7.6±0.92 ^b^ | 7.1±0.83 ^b^ | 6.6±0.66 ^b^ | 7.8±0.87 ^b^ | 7.3±0.9 ^b^ |
| Sour taste | 1.3±1.1 ^a^ | 6.6±0.8 ^c^ | 4.6±0.8 ^b^ | 7±1.1 ^c^ | 4.5±0.67 ^b^ | 4.8±0.75 ^b^ |
| Fruit flavor | 1.5±1.36 ^a^ | 6.9±0.94 ^d^ | 6.5±0.67 ^d^ | 6.4±1.11 ^cd^ | 5±0.77 ^b^ | 5.2±0.75 ^bc^ |
| Total | 11.8±2.79 ^a^ | 43.2±1.78 ^d^ | 38±2.37 ^bc^ | 40.5±1.28 ^cd^ | 37.4±2.33 ^bc^ | 37.3±2.1 ^b^ |

Each row is compared without the same letter indicating P < 0.05

**Table S3** Changes of relative mass concentration of volatile flavor compounds before and after fermentation

| **No.** | **RT**  **min** | **CAS #** | **Volatile Components** | **Relative mass concentration（μg·L^-1^）** | |
| --- | --- | --- | --- | --- | --- |
|  |  |  |  | **Before fermentation** | **After fermentation** |
|  |  |  | Ester compounds |  |  |
| 1 | 2.38 | 141-78-6 | Ethyl Acetate | 3.66 | - |
| 2 | 25.1 | 134-20-3 | Methyl anthranilate | 7.65 | 3.89 |
| 3 | 26.89 | 85-91-6 | Methyl methylanthranilate | 323.84 | 221.00 |
| 4 | 28.14 | 103-54-8 | 2-Propen-1-ol,3-phenyl-, 1-acetate | 9.01 | 4.68 |
| 5 | 30.32 | 17092-92-1 | (2,6,6-Trimethyl-2-hydroxycyclohexylidene)acetic acid lactone | - | 1.34 |
| 6 | 30.41 | 109-20-6 | Geranyl isovalerate | - | 0.56 |
| 7 | 30.71 | 57156-91-9 | 2,5-Octadecadiynoic acid, methyl ester | - | 0.48 |
| 8 | 34.4 | 120-51-4 | Benzyl Benzoate | 6.14 | 4.32 |
|  |  | Subtotal | 8 | 5 | 7 |
|  |  |  | Alkene compounds |  |  |
| 9 | 15.08 | 586-62-9 | Cyclohexene,1-methyl-4-(1-methylethylidene)- | 2.53 | - |
| 10 | 15.34 | 460-01-5 | (3E,5E)-2,6-Dimethyl-1,3,5,7-octatetrene | 11.13 | - |
| 11 | 18.91 | 6090-09-1 | 4-acetyl-1-methyl-1-cyclohexene | 12.18 | 2.68 |
| 12 | 19.11 | 1195-92-2 | limonene 1,2-epoxide | 53.14 | - |
|  |  | Subtotal | 4 | 4 | 1 |
|  |  |  | Ketone compounds |  |  |
| 13 | 3.38 | 6137-11-7 | 4-methylheptan-3-one | - | 1.97 |
| 14 | 7.54 | 644-78-0 | 2-Hydroxychalcone | - | 0.66 |
| 15 | 10.46 | 110-43-0 | 2-Heptanone | 7.11 | 5.09 |
| 16 | 14.03 | 110-93-0 | 5-Hepten-2-one, 6-methyl- | 45.51 | - |
| 17 | 16.77 | 98-86-2 | Acetophenone | 4.84 | - |
| 18 | 16.95 | 38284-27-4 | 3,5-Octadien-2-one | 18.68 | - |
| 19 | 17.53 | 15932-80-6 | Cyclohexanone, 5-methyl-2-(1-methylethylidene)- | 2.54 | - |
| 20 | 17.74 | 30086-02-3 | 3,5-Octadien-2-one, (E,E)- | 6.34 | - |
| 21 | 19.42 | 464-49-3 | (R)-camphor | 246.68 | 109.10 |
| 22 | 19.89 | 30460-92-5 | Pinocarvone | - | 1.75 |
| 23 | 20.05 | 528-21-2 | Gallacetophenone | 6.31 | - |
| 24 | 21.19 | 5948-04-9 | Cyclohexanone,2-methyl-5-(1-methylethenyl)-, (2R,5R)-rel- | 79.31 | 29.30 |
| 25 | 22.36 | 99-49-0 | Carvone | 38.25 | 20.53 |
| 26 | 22.65 | 89-81-6 | 2-Cyclohexen-1-one,3-methyl-6-(1-methylethyl)- | 9.39 | 12.27 |
| 27 | 23.08 | 16750-82-6 | (S)-3-Methyl-6β-isopropenyl-2-cyclohexene-1-one | - | 2.61 |
| 28 | 25.05 | 491-09-8 | 2-Cyclohexen-1-one, 3-methyl-6-(1-methylethylidene)- | 5.07 | - |
| 29 | 26.1 | 23696-85-7 | 2-Buten-1-one, 1-(2,6,6-trimethyl-1,3-cyclohexadien-1-yl)- | - | 4.10 |
| 30 | 29.09 | 14901-07-6 | 3-Buten-2-one, 4-(2,6,6-trimethyl-1-cyclohexen-1-yl)- | - | 3.08 |
| 31 | 29.11 | 79-77-6 | β-Ionone | 1.82 | - |
| 32 | 29.18 | 23267-57-4 | 3-Buten-2-one, 4-(2,2,6-trimethyl-7-oxabicyclo[4.1.0]hept-1-yl)- | 2.62 | 1.01 |
|  |  | Subtotal | 20 | 14 | 12 |
|  |  |  | Acid compounds |  |  |
| 33 | 2.44 | 64-19-7 | Acetic acid | - | 123.78 |
| 34 | 14.45 | 142-62-1 | Hexanoic acid | - | 14.36 |
| 35 | 17.44 | 111-14-8 | Heptanoic acid | - | 1.71 |
| 36 | 18.49 | 1783-84-2 | 8,11,14-Eicosatrienoic acid, (Z,Z,Z)- | - | 0.60 |
| 37 | 18.6 | 1883-13-2 | Dodecanoic acid, 3-hydroxy- | - | 0.55 |
| 38 | 19.97 | 7333-25-7 | 10,12-Octadecadiynoic acid | - | 1.05 |
| 39 | 29.56 | 2507-55-3 | Tetradecanoic acid, 2-hydroxy- | - | 0.61 |
|  |  | Subtotal | 7 | 0 | 7 |
|  |  |  | Aldehyde compounds |  |  |
| 40 | 2.89 | 590-86-3 | Butanal, 3-methyl- | 11.83 | - |
| 41 | 3.02 | 96-17-3 | Butanal, 2-methyl- | 7.26 | - |
| 42 | 3.61 | 110-62-3 | Pentanal | 17.88 | - |
| 43 | 5.28 | 1576-87-0 | 2-Pentenal, (E)- | 2.73 | - |
| 44 | 6.35 | 107-86-8 | 2-Butenal, 3-methyl- | - | 1.43 |
| 45 | 6.48 | 5204-80-8 | 4-Pentenal, 2-ethyl- | - | 0.28 |
| 46 | 6.94 | 66-25-1 | Hexanal | 172.42 | - |
| 47 | 8.19 | 98-01-1 | Furfural | 4.05 | - |
| 48 | 9.04 | 6728-26-3 | 2-Hexenal, (E)- | 9.83 | - |
| 49 | 10.94 | 111-71-7 | Heptanal | 7.58 | - |
| 50 | 13.12 | 100-52-7 | Benzaldehyde | 266.16 | 13.03 |
| 51 | 14.99 | 4313-03-5 | 2,4-Heptadienal, (E,E)- | 5.37 | - |
| 52 | 16.05 | 122-78-1 | Benzeneacetaldehyde | 13.26 | - |
| 53 | 18.1 | 124-19-6 | Nonanal | 10.16 | - |
| 54 | 19.9 | 104-53-0 | Benzenepropanal | 49.55 | - |
| 55 | 21.43 | 99172-18-6 | 3,5-Heptadienal, 2-ethylidene-6-methyl- | - | 0.36 |
| 56 | 21.51 | 15764-16-6 | Benzaldehyde, 2,4-dimethyl- | 113.29 | 23.27 |
| 57 | 23.25 | 104-55-2 | Cinnamaldehyde | 3402.57 | 7.55 |
| 58 | 30.43 | 1504-74-1 | 2-Propenal, 3-(2-methoxyphenyl)- | 10.11 | - |
| 59 | 34.19 | 1620-98-0 | 3,5-di-tert-Butyl-4-hydroxybenzaldehyde | - | 0.98 |
|  |  | Subtotal | 20 | 16 | 7 |
|  |  |  | Phenolic compounds |  |  |
| 60 | 23.83 | 89-83-8 | Thymol | 26.29 | 37.05 |
| 61 | 29.71 | 128-37-0 | Butylated Hydroxytoluene | 22.02 | 24.40 |
|  |  | Subtotal | 2 | 2 | 2 |
|  |  |  | Alcohol compounds |  |  |
| 62 | 1.48 | 64-17-5 | Ethanol | 16.73 | 71.57 |
| 63 | 3.35 | 616-25-1 | 1-Penten-3-ol | 16.46 | - |
| 64 | 4.69 | 123-51-3 | 1-Butanol, 3-methyl- | 13.57 | - |
| 65 | 5.82 | 71-41-0 | 1-Pentanol | 8.43 | - |
| 66 | 5.9 | 1576-95-0 | 2-Penten-1-ol, (Z)- | 7.03 | - |
| 67 | 9.79 | 111-27-3 | 1-Hexanol | 10.78 | - |
| 68 | 10.01 | 111-70-6 | 1-Heptanol | 0.50 | - |
| 69 | 10.1 | 928-96-1 | 3-Hexen-1-ol, (Z)- | 3.89 | 2.22 |
| 70 | 10.75 | 928-97-2 | 3-Hexen-1-ol, (E)- | - | 0.57 |
| 71 | 10.84 | 6728-31-0 | 4-Heptenal, (Z)- | 3.89 | - |
| 72 | 10.94 | 51174-44-8 | 4-Penten-1-ol, 3-methyl- | - | 0.70 |
| 73 | 11.4 | 626-89-1 | 1-Pentanol, 4-methyl- | - | 0.48 |
| 74 | 14.56 | 3391-86-4 | 1-Octen-3-ol | 65.05 | 0.67 |
| 75 | 14.68 | 99-48-9 | Carveol | 20.75 | - |
| 76 | 17.51 | 5989-33-3 | 2-Furanmethanol,5-ethenyltetrahydro-a,a,5-trimethyl-, (2R,5S)-rel- | - | 10.85 |
| 77 | 17.98 | 78-70-6 | Linalool | 479.86 | 229.34 |
| 78 | 18.69 | 7212-40-0 | 2-Cyclohexen-1-ol, 1-methyl-4-(1-methylethenyl)-, trans- | 25.34 | 6.59 |
| 79 | 18.73 | 29803-81-4 | 2-Cyclohexen-1-ol, 1-methyl-4-(1-methylethyl)-, trans- | 9.13 | - |
| 80 | 19.17 | 3886-78-0 | cis-p-Mentha-2,8-dien-1-ol | 1.58 | 6.17 |
| 81 | 19.59 | 6627-74-3 | 2-Cyclohexene-1-methanol, 2,6,  6-trimethyl- | - | 2.51 |
| 82 | 20.25 | 507-70-0 | endo-Borneol | 25.59 | - |
| 83 | 20.49 | 562-74-3 | Terpinen-4-ol | 574.29 | 279.83 |
| 84 | 20.74 | 1197-01-9 | Benzenemethanol,α,α,4-trimethyl- | 31.39 | 10.50 |
| 85 | 20.98 | 98-55-5 | α-Terpineol | 607.59 | 310.14 |
| 86 | 22.52 | 1197-06-4 | 2-Cyclohexen-1-ol, 2-methyl-5-(1-methylethenyl)-, (1R,5R)-rel- | 119.21 | 16.58 |
| 87 | 25.59 | 104-54-1 | Cinnamyl alcohol | - | 12.87 |
| 88 | 31.42 | 6750-60-3 | Spathulenol | 25.58 | 11.59 |
| 89 | 32.56 | 117591-80-7 | Ginsenol | - | 0.86 |
|  |  | Subtotal | 28 | 21 | 18 |
|  |  |  | Other compounds |  |  |
| 90 | 17.59 | 1124-20-5 | 1-methyl-3-prop-1-en-2-ylbenzene | - | 6.39 |
| 91 | 28.6 | 719-22-2 | 2,5-Cyclohexadiene-1,4-dione, 2,6-bis(1,1-dimethylethyl)- | 4.02 | - |
|  |  | Subtotal | 2 | 1 | 1 |
|  |  | Total | 91 | 63 | 55 |

“-” means not detected.
